# Calcium Binding and Disulfide Bonds Regulate the Stability of Secretagogin towards Thermal and Urea Denaturation

**DOI:** 10.1371/journal.pone.0165709

**Published:** 2016-11-03

**Authors:** Kalyani Sanagavarapu, Tanja Weiffert, Niamh Ní Mhurchú, David O’Connell, Sara Linse

**Affiliations:** 1 Department of Biochemistry and Structural Biology, Lund University, Lund, Sweden; 2 School of Biomolecular and Biomedical Science, Conway Institute, University College Dublin, Dublin, Ireland; Universitat Politecnica de Catalunya, SPAIN

## Abstract

Secretagogin is a calcium-sensor protein with six EF-hands. It is widely expressed in neurons and neuro-endocrine cells of a broad range of vertebrates including mammals, fishes and amphibia. The protein plays a role in secretion and interacts with several vesicle-associated proteins. In this work, we have studied the contribution of calcium binding and disulfide-bond formation to the stability of the secretagogin structure towards thermal and urea denaturation. SDS-PAGE analysis of secretagogin in reducing and non-reducing conditions identified a tendency of the protein to form dimers in a redox-dependent manner. The denaturation of apo and Calcium-loaded secretagogin was studied by circular dichroism and fluorescence spectroscopy under conditions favoring monomer or dimer or a 1:1 monomer: dimer ratio. This analysis reveals significantly higher stability towards urea denaturation of Calcium-loaded secretagogin compared to the apo protein. The secondary and tertiary structure of the Calcium-loaded form is not completely denatured in the presence of 10 M urea. Reduced and Calcium-loaded secretagogin is found to refold reversibly after heating to 95°C, while both oxidized and reduced apo secretagogin is irreversibly denatured at this temperature. Thus, calcium binding greatly stabilizes the structure of secretagogin towards chemical and heat denaturation.

## Introduction

Secretagogin is a hexa EF-hand calcium binding protein that is widely expressed in many tissues. The protein is found in multiple neuronal subtypes in the brain, pancreatic β-cells of the gastro intestinal tract and erythrocytes [1–4]. Recent studies reveal that the expression pattern of secretagogin in brain differs among human, mouse and rat. In human brain the protein is most highly expressed in the cerebellum whereas in mouse and rat the highest expression of secretagogin is seen in the olfactory bulb [3, 5]. Secretagogin exists in three different forms, two of which are characterized by a single amino acid difference [glutamine (Q)/arginine (R)] at residue 22 (secretagogin Q-22 and secretagogin R-22). The third form, setagin, is an alternatively spliced form of secretagogin and consists of only 49 amino acids. Setagin does not bind Ca^2+^, with the introduction of a stop codon after residue 49 it has lost all the EF-hand motifs [6].

**Fig 1:**
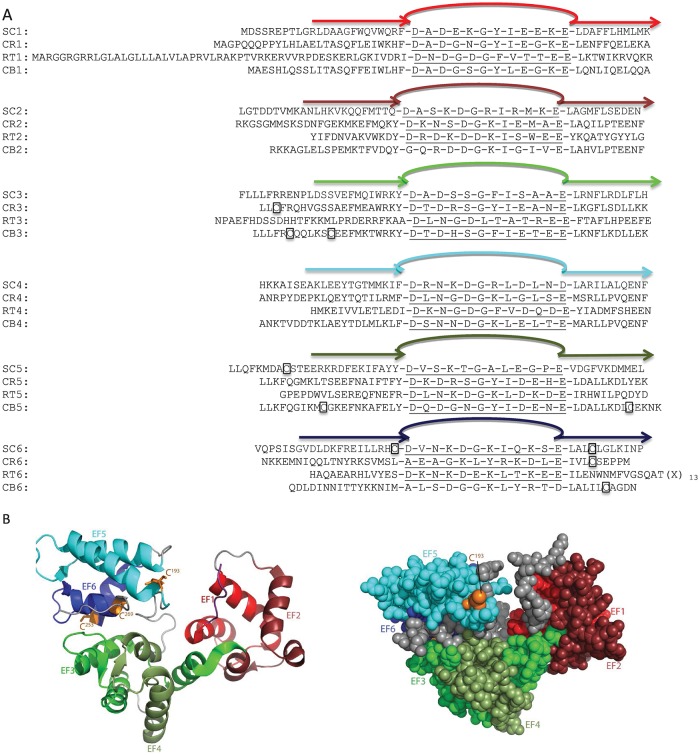
(A) Alignment of EF-hands of human secretagogin (SC) and the homologues calretinin (CR), reticulo calbin (RT) and calbindin (CB). The six EF-hands are labelled in different colors (red: EF1, dark red: EF2, green: EF3, pale green: EF4, cyan: EF5 and dark blue: EF6. Cysteines are boxed. (B) Crystal structure of secretagogin from *Danio rerio* (zebra fish), PDB ID: 2BE4. Six EF hands are colored as above. Cysteines are shown in orange, C193 is visible on the front surface, while C253 and C269 are on the back side. RT6: (X)_13_ = NYGEDLTKNHDEL.

**Fig 2:**
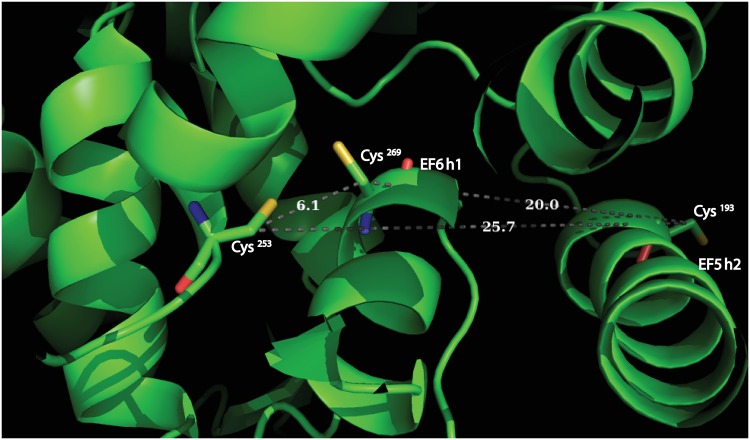
Illustration of pairwise Cys-Cys distance in human secretagogin structure mapped on *Dan rerio* secretagogin X-ray structure, PDB ID: 2BE4.

Full length secretagogin is a protein of 276 amino acid residues with a calculated molecular mass of 32 kDa. Multiple sequence alignments over mammalian secretagogins show a sequence identity of 70–80% [7]. The six EF-hands are helix-loop-helix sub domains [8] and the protein undergoes calcium induced conformational changes [4]. Secretagogin can bind up to four Ca^2+^ ions with high affinity [9, 10], thus two of the EF-hands have significantly lower affinity. The human secretagogin has three cysteines- C^193^, C^253^ and C^269^. C^253^ and C^269^ are conserved in rat, mouse, pig, and zebra fish. Four different disulfide linkages have been identified from MS/MS spectra of recombinant human secretagogin [11]. C^193^- C^193^ and C^269^- C^269^ are inter-protein and C^193^-C^269^ and C^253^-C^269^ are intra-protein linkages. A 2.1-Å resolution X-ray crystal structure is known for zebra fish secretagogin (Fig 1) which is 73% identical to human secretagogin [7]. The structure reveals that residues 253 and 269 (Cβ-Cβ = 6.1 Å), are close enough to form disulfide bonds with little rearrangement, whereas 193 and 269 (Cβ-Cβ = 20 Å) and 193 and 253 (Cβ-Cβ = 25.7 Å) are too far apart. Formation of the disulfide bond between Cys 193 and 269 [11] would require conformational changes relative to the zebra fish X-ray structure [7] e.g. rotation of helices EF5 h2 and EF6 h1 (Fig 2). The structure further reveals a heart-shaped protein in which EF-hands 1 and 2 are in one lobe, EF-hands 3 and 4 at the tip of the heart and EF-hands 5 and 6 in the other lobe. Between the lobes there is a deep cleft, but it is not known whether this is the binding site for target proteins.

Many EF-hand proteins behave either as signaling proteins or buffering proteins. Ca^2+^-buffers or transporters have high Ca^2+^ affinities (*K*_D_ <0.1 *μ*M) and undergo only minor conformational changes upon binding of Ca^2+^ [4]. Ca^2+^-buffer proteins function to maintain nontoxic levels of free intracellular Ca^2+^. Ca^2+^ sensors mediate signaling cascades in response to changes in calcium concentration. They have lower Ca^2+^ affinities (*K*_D_ >1 *μ*M) and undergo significant Ca^2+^-induced conformational changes, which result in exposure or modulation of binding surfaces for target proteins. The target proteins are often activated or attenuated upon complex formation. For example, the Ca^2+^ sensor protein calmodulin may activate a large number of protein kinases that initiate cytoplasmic and nuclear processes [12, 13]. These processes are tightly controlled by the Ca^2+^ concentration in the cell and the calcium binding affinity of the EF-hand protein [12].

Secretagogin has a distinct set of ten known targets that are mostly vesicular proteins, but also some cytosolic enzymes [9, 14–18]. The reported targets are SNAP25 involved in Ca^2+^-induced exocytosis [9], SNAP23, DOC2alpha, ARFGAP2, rootletin, KIF5B, β-tubulin, DDAH-2, ATP-synthase and myeloid leukemia factor 2 [14] as well as the 4R isoform of Tau [18]. Secretagogin function as a Ca^2+^ sensor has been investigated in a range of physiological processes. Studies of secretagogin function in insulin synthesis and secretion [10] have revealed an increase in expression of secretagogin in pancreatic β-cells in response to insulin [18]. Most recently, a putative role for secretagogin in the release of corticotrophin-releasing hormone by parvocellular neurons of the hypothalamic paraventricular nucleus in stress responsiveness has been described [19].

The closest homologues of secretagogin are the hexa EF-hand proteins calbindin D28k, calretinin and reticulocalbin (Fig 1), whereas more distant homologues troponin c, calmodulin, parvalbumin, calbindin D9k, etc. contain fewer EF-hands. Stability studies of calbindin D28k and calretinin have been reported [20, 21].

In this study, we have investigated the stability of secretagogin towards urea and thermal denaturation, in the absence and presence of Ca^2+^, and in the reduced and oxidized states. The structural changes occurring upon denaturation were monitored using circular dichroism and fluorescence spectroscopy. The tendency of secretagogin to form dimers was examined using SDS-PAGE analysis. These studies can help us understand the factors that govern the native fold of this protein and its response to calcium binding and oxidation.

## Results

### Disulfide-dependent secretagogin dimers

**Fig 3:**
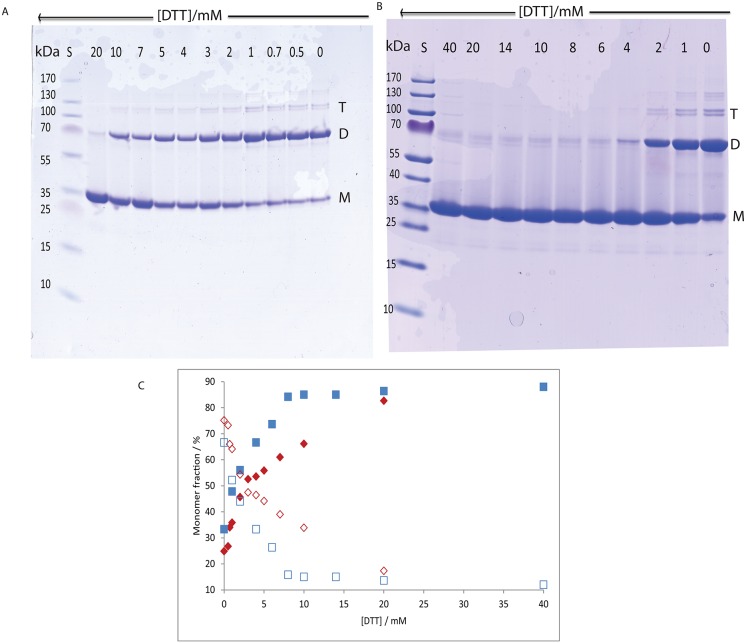
SDS-PAGE analysis of secretagogin dimers. (3A) Non-reducing gel of apo secretagogin. (3B) Non-reducing gel of calcium-loaded secretagogin. Lane 1 has pre stained protein ladder. Lanes 2–12 have 1 mg/mL secretagogin (30 μM) with 20 mM DTT, 10 mM DTT, 7 mM DTT, 5 mM DTT, 4 mM DTT, 3 mM DTT, 2 mM DTT, 1 mM DTT, 0.7 mM DTT (not present in 3B), 0.5 mM DTT and no DTT respectively. M = monomer; D = dimer; T = trimer (3C) Percent of monomer versus concentrations of DTT and dimer versus DTT concentration. Red: apo protein; blue: calcium-loaded protein; filled box: monomer; non-filled box: dimer.

**Fig 4:**
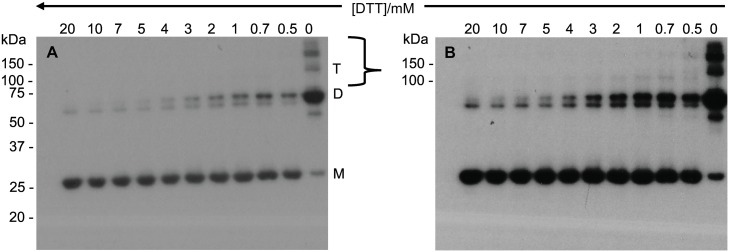
Anti secretagogin Western blot analysis of recombinant secretagogin oligomers. Non-reducing Western blot of apo secretagogin exposed for 2 min (4A) and 5 min (4B) with Pierce^™^ chemiluminescent Western blotting substrate. Lane 1–11 has 2 μg of 1 mg/mL purified secretagogin with 20 mM DTT, 10 mM DTT, 7 mM DTT, 5 mM DTT, 4 mM DTT, 3 mM DTT, 2 mM DTT, 1 mM DTT, 0.7 mM DTT, 0.5 mM DTT and no DTT respectively. M = monomer; D = dimer; T = trimer.

SDS-PAGE was used to study the monomer: dimer distribution of apo and calcium-loaded secretagogin as a function of DTT concentration (0–20 mM, Fig 3A and 3B). The DTT used while purifying secretagogin was removed by dialysis, before defined concentrations of DTT were added and the SDS-PAGE was run after 16 h incubation. The intensity of the bands on a Coomassie-stained gel was analyzed using ImageJ software and plotted as the percentage of monomer versus DTT concentration (Fig 3C). In the absence of DTT, ca. 25% of the apo protein is present as monomers and 75% as dimers while calcium-loaded protein contains ca. 33% as monomer and 67% as dimer. In the presence of DTT, the apo protein progressively shifted towards monomer the higher the DTT concentration (Fig 3C). At the highest DTT concentration, 20 mM, a very faint band for dimer is seen. This result implies that dimer formation is due to disulfide bond formation. The monomer form of the calcium-loaded protein is favored above 0.5 mM and there is ca. 85% as monomer at 4 mM DTT and higher. For apo secretagogin, the monomer dominates at DTT concentrations above 3 mM, thus the disulfide bond in apo secretagogin is more resistant to reduction by DTT. This is confirmed by Western blot using an anti-secretagogin antibody (Fig 4).

This analysis shows that a molar ratio of ca. 670 DTT: apo secretagogin (20 mM DTT: 30 μM apo secretagogin) is needed to maintain the protein in a predominantly monomeric form. Approximately 54% of monomer and 46% dimer are observed at 4 mM DTT (molar ratio 170). A molar ratio of ca. 170 DTT: calcium-loaded protein (4 mM DTT: 30 μM calcium-loaded secretagogin) is needed to maintain the protein in predominantly monomeric form. At the protein concentration used in denaturation studies, 6.0 μM, the higher ratio (670) corresponds to 4 mM DTT, and the lower ratio (180) to 1 mM DTT. The same result was obtained with freshly prepared protein and protein that had been stored at -20°C after purification (S1 and S2 Figs).

**Fig 5:**
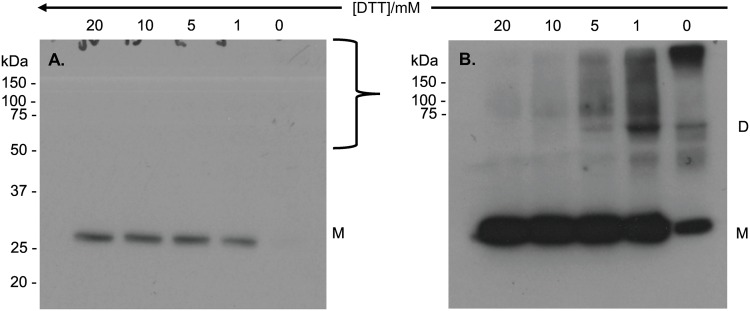
Anti secretagogin Western blot analysis of endogenous secretagogin in BRIN-BD11 insulinoma cell lysates at selected concentrations of DTT. **(5A)** Non-reducing Western blot of native secretagogin exposed for 30 s with low sensitivity Pierce^™^ chemiluminescent Western blotting substrate (Thermo Scientific, cat. no. 32106). (5B) Non-reducing Western blot of native secretagogin exposed for 5 min with high sensitivity BM chemiluminescent Western blotting substrate (Roche, cat. no. 11500708001). Each lane has 30 μg of total protein with 20 mM DTT, 10 mM DTT, 5 mM DTT, 1 mM DTT, and no DTT respectively. M = monomer; D = dimer.

**Fig 6:**
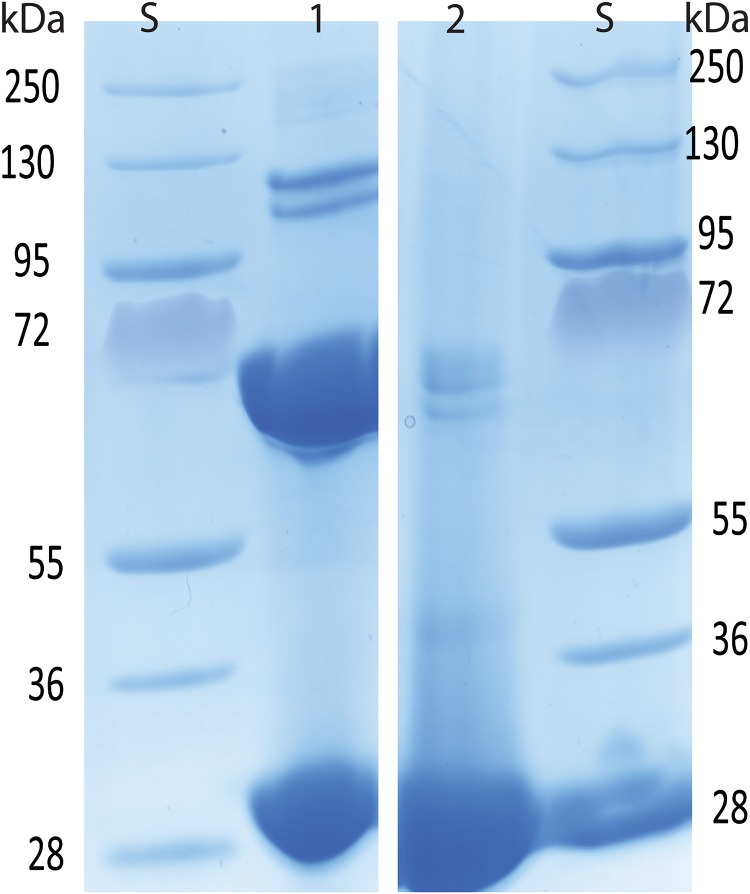
*Lane* 1: Apo secretagogin reduced in 20 mM DTT and dialyzed in glutathione redox buffer of -170 mV potential. Lane 2: Apo secretagogin reduced in 20 mM DTT.

SDS-PAGE and Western blotting was used to investigate whether the monomer: dimer distribution of secretagogin purified after overexpression in *Escherichia coli* BL21 Star, as a function of DTT concentration (0–20 mM, Fig 3A), was comparable with that of the BRIN-BD11 rat clonal pancreatic β-cell line. Wagner *et* al [10] have previously shown an abundant expression of secretagogin in the pancreas using rat RIN-5F insulinoma cells, a cell line from which BRIN-BD11 is derived [22]. Here we used Western blot analysis of samples at selected DTT concentrations (20 mM, 10 mM, 5 mM, 1 mM, 0 mM) with a chemiluminescent-based detection method and two reagents with different sensitivities (Fig 5). Irrespective of exposure time, only monomeric protein is observed at 1–20 mM DTT with the least sensitive reagent (~1 fmol detection limit) (Fig 5A). Monomeric secretagogin is undetectable in the absence of DTT. Dimer and higher species are detected using the 10-fold more sensitive reagent at 0–5 mM DTT, but not at 10–20 mM DTT. Furthermore, higher oligomeric species appear above the 150 kDa protein standard only in the absence of DTT (Fig 5B). The apparent ratio of secretagogin monomer: dimer is thus different in purified recombinant secretagogin and BRIN-BD11 cell sample. The redox potential of insulinoma cells is low. This could be a reason why a major fraction of monomer is observed in insulinoma cell lysates. Whereas in the study of pure protein (Fig 3) the redox potential was varied, showing dimer formation under more oxidizing conditions. To test this *E*.*coli* purified secretagogin is incubated overnight with GSSG+GSH redox buffer of -170 mV to check if a reverse effect on oligomerization can be seen as compared to DTT reduction. Most of the protein is seen as dimer and higher oligomers as analyzed by SDS-PAGE (Fig 6).

### Urea induced unfolding of secretagogin

**Fig 7:**
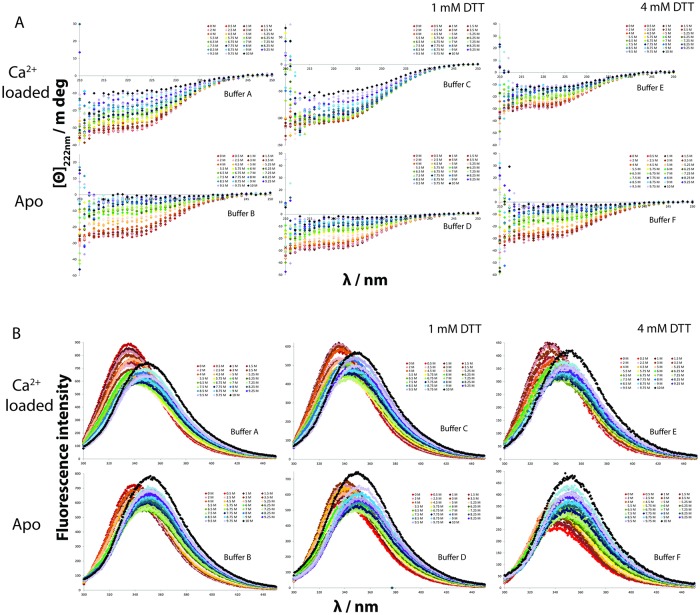
Urea induced denaturation of secretagogin studied in non-reducing conditions (10 mM Tris, 0.15 M KCl [A, D] with 1 mM CaCl_2_ or 0.5 mM EDTA. pH: 7.5) and reducing conditions (10 mM Tris, 0.15 M KCl, 1mM [B, E] or 4 mM DTT [C, F] with 1 mM CaCl_2_ or 0.5 mM EDTA, pH: 7.5) using (7A) circular dichroism spectroscopy and (7B) fluorescence spectroscopy. [Θ]_222 nm_ is the ellipticity at 222 nm; λ is the wavelength; Panel A shows [Θ]_222 nm_ versus λ as measured using CD spectroscopy and panel B shows fluorescence intensity versus λ as measured using fluorescent spectrometer.

**Fig 8:**
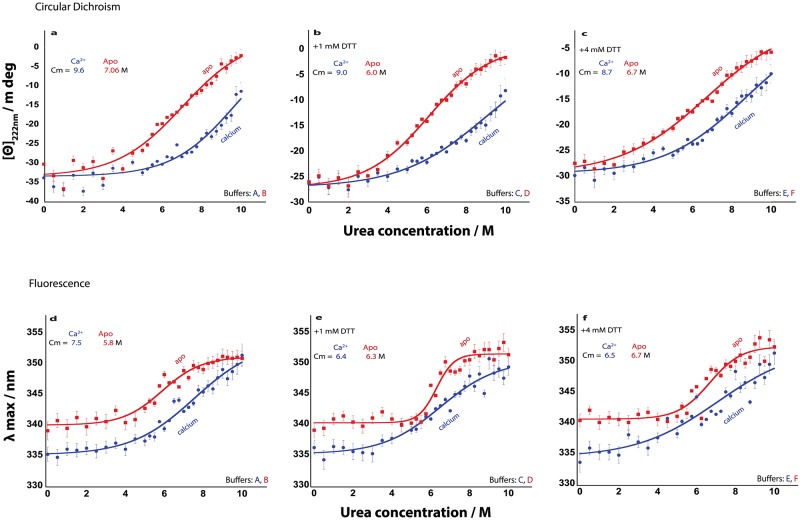
Urea induced denaturation of secretagogin in non-reducing conditions (10 mM Tris, 0.15 M KCl [A, D] with 1 mM CaCl_2_ or 0.5 mM EDTA. pH: 7.5) and reducing conditions (10 mM Tris, 0.15 M KCl, 1mM [B, E] or 4 mM DTT [C, F] with 1 mM CaCl_2_ or 0.5 mM EDTA, pH: 7.5). Blue and red curves represent calcium-loaded and apo form of secretagogin, respectively. [Θ]_222 nm_ is the ellipticity at 222 nm; λ max is the wavelength at which the fluorescent intensity is highest; Cm is the urea concentration at the transition midpoint. In panels a-c are shown [Θ]_222 nm_ versus urea concentration and in panels d-f λ max versus urea concentration.

The stability of secretagogin towards urea denaturation was studied at reducing and non-reducing conditions. The samples containing 670 molar equivalents DTT are referred to as reduced and samples without DTT as non-reduced, while 170 equivalents was used as an intermediate condition with close to half reduction. The secondary structure of secretagogin was followed by CD spectroscopy, and the unfolding of helices was monitored by the ellipticity at 222 nm as a function of urea concentration (Fig 7A). The tertiary structure of protein was monitored by fluorescence spectroscopy and the change in the Trp fluorescence intensity upon unfolding was reported (Fig 7B). The signal versus urea concentration follows a sigmoidal curve by both methods, reflecting the unfolding of the protein secondary and tertiary structure. This is seen in both reducing and non-reducing conditions (Fig 8). A pre-transition baseline is followed by a sharper transition for the apo form and a shallower transition for the calcium-loaded form. A distinct post-transition plateau is observed for apo secretagogin in the presence of DTT, when followed by fluorescence spectroscopy, but is short or absent in the other curves. The apo secretagogin curve displays a transition at lower urea concentration than the calcium form indicating that it is less stable than the calcium form.

The data were fitted using Eq 1 and the apparent values of the free energy of unfolding, ΔG_NU_ (H_2_O), in the absence of urea, and C_m_, the urea concentration at the transition mid-point are shown in the respective panels. The data are thus well fitted assuming a two-state model with no intermediates. The concentration of urea at transition mid-point (C_m_) of both apo and calcium secretagogin is found to vary only slightly, by maximum 1.0 M urea over the DTT concentrations tested (Fig 8). For both apo and calcium-bound secretagogin, the denaturation appears to be redox-independent. Moreover, the C_m_ values show much higher tolerance of calcium-loaded secretagogin compared to apo secretagogin towards urea denaturation by maximum 3 M units. At no condition does the calcium-loaded protein appear to be fully denatured at 10 M urea, implying a highly stable protein.

### Temperature denaturation of secretagogin

**Fig 9:**
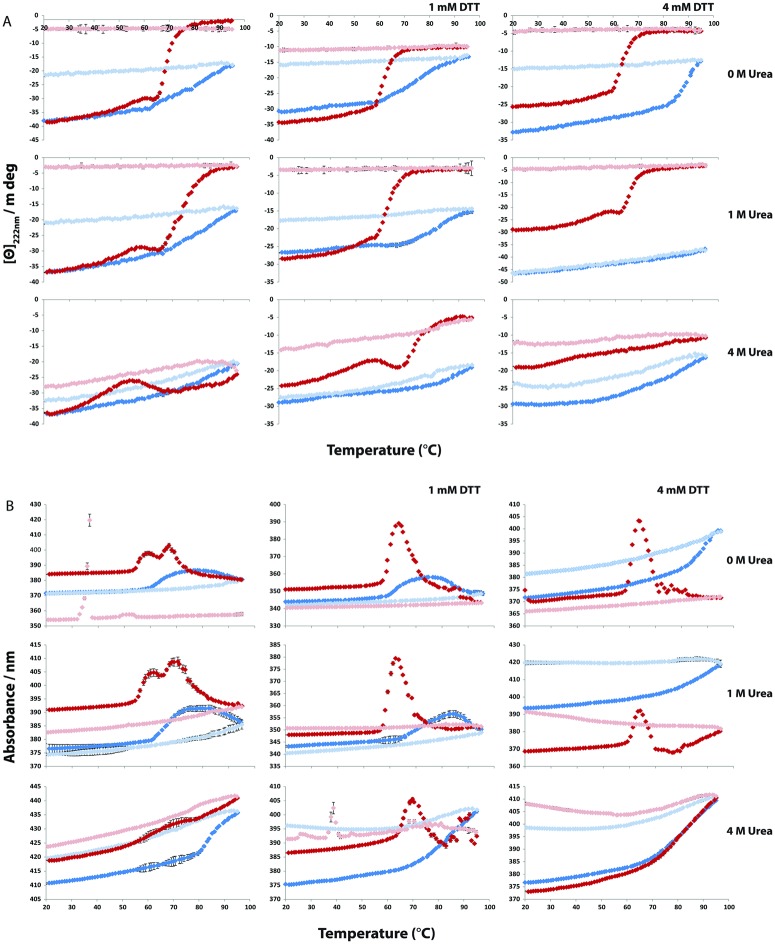
Thermal stability of secretagogin as analyzed by circular dichroism spectroscopy when heated from 20 to 95°C in six buffers, A-F (Table 3). [Θ]_222 nm_ is ellipticity at 222 nm. Blue and pale blue dots represent calcium-loaded secretagogin under heating (20–95°C) and cooling (95–20°C), respectively. Red and pale red dots represent apo secretagogin under heating (20–95°C) and cooling (95–20°C), respectively. A. [Θ]_222 nm_ versus temperature. B. Total absorbance (nm) versus temperature.

The thermal stability of the calcium and apo form of secretagogin was studied using CD spectroscopy in the presence or absence of urea or DTT. Curves with a sigmoidal transition and distinct pre- and post-transition baselines are seen for apo secretagogin at 0 and 1.0 M urea with and without DTT (Fig 9) although some curves display more than one transition. This suggests that intermediate may exist along the thermal denaturation pathway, at least under some conditions. At 4 M urea, the apo samples display a hump followed by a sigmoidal transition. Under all conditions, thermal denaturation of apo secretagogin appears to be irreversible as the signal is not regained upon cooling (Fig 6). In the presence of calcium, a sigmoidal transition with distinct pre-transition baseline is observed at 0 and 1.0 M urea and 0 or 1 mM DTT. At 4 mM DTT and no urea, the stability towards thermal denaturation is very high and the transition is just starting around 90–95°C. Again, the process is irreversible. However, in the presence of 4.0 M urea and 0–4 mM DTT, or 1.0 M urea and 4 mM DTT, the process is reversible. Because of the lack of reversibility in most cases, or lack of post—transition baseline in other cases, T_m_ values could not be determined. Therefore, the data were used to estimate apparent T_m_ values, which are still interesting to compare as they reflect the relative resistance to denaturation under the present procedure (scan rate 1°C/min) (Table 1a) and can be used to compare behavior in different solution conditions.

**Table 1:** Summary of apparent Tm and the real reversibility temperature range of secretagogin in various conditions studied.

Urea concentration	No DTT		1 mM DTT		4 mM DTT	
	apo	calcium-loaded	apo	calcium-loaded	apo	calcium-loaded
**1a.Apparent Tm / °C**						
0 M	62	76	61.5	75	65	90
1 M	63	78	64	79	66	>95
4 M	63	83	64	86	70	>95
**1b.Reversibility range/ °C**						
0 M	20–45	20–55	20–50	20–65	20–55	20–65
1 M	20–45	20–55	20–50	20–65	20–55	20–95
4 M	20–55	20–60	20–60	20–65	20–65	20–95

We also examined the total absorbance / turbidity recorded by the CD spectrometer at 222 nm. These absorbance curves showed distinct features in similar temperature ranges as the ellipticity curves. In the absence of DTT where apo secretagogin displayed more than one transition, the absorbance curves displayed two peaks although at 4.0 M urea the second peak is not distinct. All the absorption curves of apo secretagogin show at least one peak and each peak corresponds to the mid-point of a transition in the ellipticity curve. We find a complete denaturation of the protein when heated to 95°C in most of the conditions. However, in the presence of 4.0 M urea and 4 mM DTT, the transition is just starting close to 90–95°C and the protein is able to refold to a significant extent upon cooling.

None of the absorption curves of calcium-loaded secretagogin showed a distinct peak, and the ellipticity does not reach any post-transition plateau, which implies that the protein is not completely denatured at 95°C, confirming that this form is highly stable. For calcium-loaded secretagogin at 0 and 1.0 M urea and 0 and 1 mM DTT, a peak seems to emerge at the high temperature end. In the other cases, a change in absorbance is just starting around 90–95°C and the protein is able to refold upon cooling.

#### Reversibility of thermal denaturation

**Fig 10:**
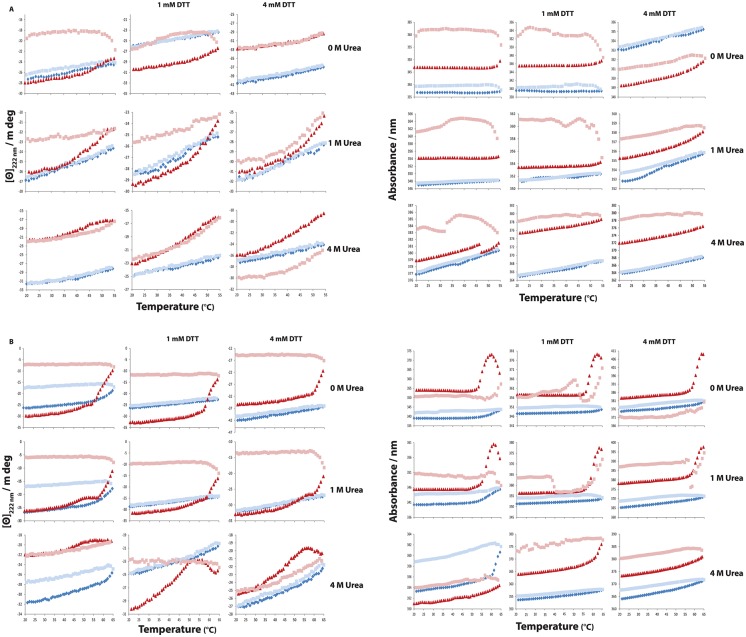
The reversibility ranges of secretagogin analyzed by circular dichroism spectroscopy. Curves here represent temperature denaturation of both apo and calcium-loaded secretagogin during heating from 20–55°C and 20–65°C and from the maximum temperature to 20°C, for apo and calcium-loaded secretagogin at 0, 1 and 4 M urea and 0, 1 and 4 mM DTT. [Θ]_222 nm_ is ellipticity at 222 nm. Blue and pale blue dots represent calcium-loaded secretagogin during heating from 20°C to test temperature and reverse scan to 20°C, respectively. Red and pale red dots represent apo secretagogin during heating from 20°C to test temperature and reverse scan to 20°C, respectively. (A) [Θ]_222 nm_ versus temperature and total absorbance (nm) versus temperature at 20–55°C. (B) [Θ]_222 nm_ versus temperature and total absorbance (nm) versus temperature obtained at 20–65°C.

In contrast to chemical denaturation, thermal denaturation appears to be irreversible (Fig 9). Tests were therefore performed to find the approximate temperature to which the protein can be heated and still reversibly return to its native fold upon cooling (Fig 10). The resulting reversibility ranges are tabulated as a function of condition (Table 1b). In the absence of DTT, apo secretagogin can refold after heating to 45°C. The presence of urea increases the reversibility range. In presence of 4M urea the apo form can refold after heating to 55°C. The calcium-loaded secretagogin in the absence of DTT can refold after heating to 55°C, while in presence of 4M urea the protein can refold even after heating to 60°C. With increasing DTT concentrations, the reversibility range is gradually increasing. For apo secretagogin the range increases by 5°C in 1 mM DTT and by 10°C in 4 mM DTT. Calcium-loaded secretagogin showed an increase of 10°C in 1 mM DTT and ≥ 10°C in 4 mM DTT. The results clearly show the greater ability of calcium-loaded secretagogin compared to apo secretagogin to refold after heating to high temperatures. Both forms display reversible denaturation in the physiological range around 37°C.

The absorption curves of both apo and calcium-loaded secretagogin at different temperatures clearly show difference in the levels of denaturation peak observed in correlation with the observed ellipticity changes. In case of apo secretagogin the peaks are just starting already above 45°C and in calcium-loaded secretagogin it is above 55°C. In the reversible temperature range, similar to urea denaturation curves, the absorption curves look almost flat with no peak incidence.

## Discussion

The results of the current study show that calcium-loaded secretagogin is a highly stable protein and that it is more stable than the apo form in both reducing and non-reducing conditions. The SDS-PAGE analysis of purified recombinant secretagogin implies that the disulfide bonds present in the protein are quite strong. A very high concentration of DTT is needed to maintain most of the protein as monomer. SDS-PAGE analysis of secretagogin in insulinoma cell lysates implies that the dimer and higher oligomer forms co-exist with monomer in the reducing environment of these cells. This would suggest that under native conditions secretagogin is roughly equally distributed between monomer and higher species. Compared to recombinant secretagogin, cellular secretagogin appears to be more sensitive to DTT, which disrupts its multimeric form, and gradually reduces the protein toward the monomeric form. Incubation of recombinant secretagogin in glutathione redox mixtures, which mimic *in vivo* redox potential (-170 mV), generated a 1:1 mixture of monomer and dimer, with some abundance of higher oligomers, which correlates well to secretagogin from native cells [20]. This again would suggest that native secretagogin is both monomeric and multimeric in cells and so the recombinant model gives a good indication of the natural state in insulinoma cells. Using cysteine substitutions may provide additional information on the influence of possible intramolecular disulfide bonds for C253/C269 and intermolecular disulfide formation for C193/C253/C269 for the stability. The results from CD spectroscopy show that apo secretagogin loses its secondary structure at lower urea concentration than the Ca^2+^- form. Also apo secretagogin denatures at lower temperatures compared to calcium-loaded secretagogin. Fluorescence data indicate that in all cases tertiary and secondary structure have the same denaturation mid-point implying a highly cooperative denaturation process.

**Fig 11:**
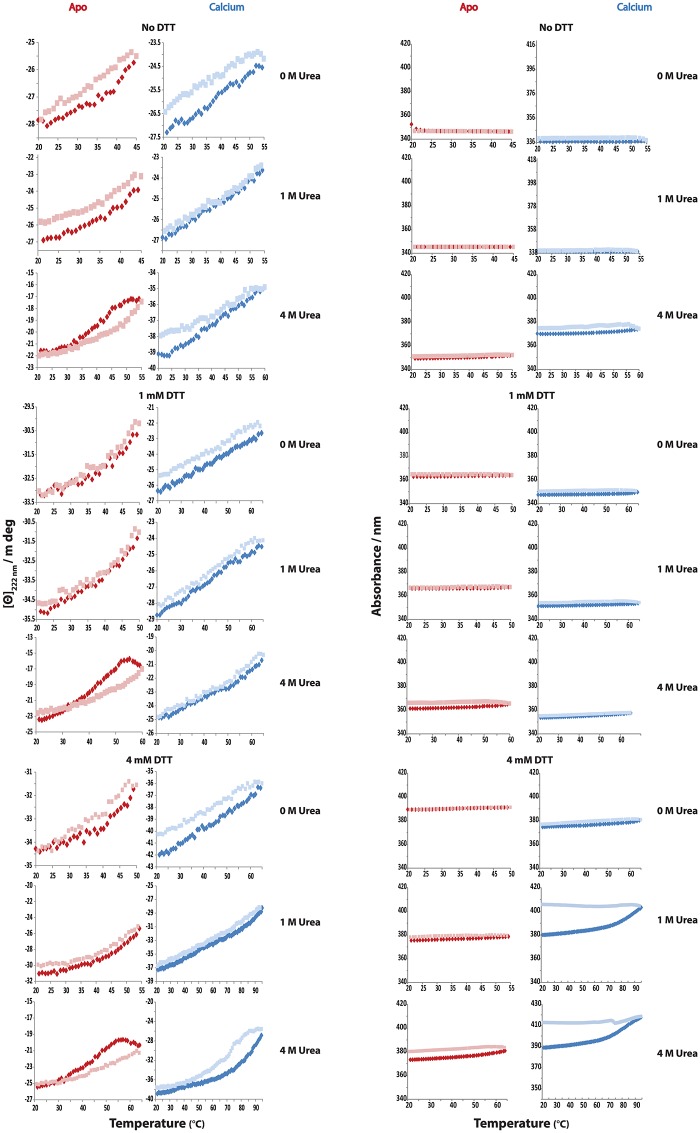
Reversible denaturation curves of secretagogin analyzed by circular dichroism spectroscopy. Secretagogin is tested for temperature reversible denaturation at different temperatures in six buffers, A-F (Table 3). The protein is heated up to the maximum temperature at each specific condition where it can refold. [Θ]_222 nm_ is ellipticity at 222 nm. Blue and pale blue dots represent calcium-loaded secretagogin heated from 20°C to test temperature and reverse scan to 20°C, respectively. Red and pale red dots represent apo secretagogin heated from 20°C to test temperature and reverse scan to 20°C, respectively. A. [Θ]_222 nm_ versus temperature. B. Total absorbance (nm) curves versus temperature.

Secretagogin appears to be highly stable towards thermal denaturation; although the process is not reversible after heating to 95°C. Calcium-loaded secretagogin is more stable than the apo form. Calcium-loaded secretagogin has an apparent Tm of 75°C in the oxidized form, which increased to 90°C upon reduction. In the presence of both urea and DTT T_m_ is >95°C. Calcium-loaded secretagogin showed reversible denaturation when heated to 55°C (non-reduced), 65°C (reduced) and 95°C (reduced and in presence of 4 M urea). The apo form has an apparent T_m_ of 62°C, which is increased by 3°C upon reduction. Moreover, the T_m_ of reduced apo protein in the presence of urea is 70°C. Reduced or non-reduced apo secretagogin is completely unfolded at 95°C in the presence or absence of urea. The process is reversible when the protein is heated to 45°C (non-reduced), 55°C (reduced) and 65°C (reduced and in presence of 4 M urea) (Fig 11). When heated to 95°C, reduced calcium-loaded secretagogin showed limited renaturation in the presence of urea. It is evident from these numbers that calcium-loaded secretagogin is more tolerant to high temperatures than apo protein in all the conditions tested. As precipitation is seen while testing temperature denaturation up to 95°C, it is impossible to derive the thermodynamic equilibrium parameters and here we only discuss apparent T_m_ values. Still secretagogin is stable at normal physiological temperature. Even the least stable form, the non-reduced apo-form, has an apparent T_m_ of 62°C and its unfolding is fully reversible up to 45°C (Fig 11). The higher stability of the reduced form implies that inter- or intra-molecular disulfide bonds are not well accommodated in the most stable secretagogin structure.

Our data show that the structure and stability of secretagogin is modulated by the solution conditions. This implies a significant degree of plasticity, which may underlie the ability of the protein to bind multiple targets involved in various functions. Several potential targets of secretagogin are involved in vesicle handling processes and in the fusion, transport and storage of vesicles. These results are highly interesting in the context of a role for secretagogin in secretion and vesicle trafficking. It remains to be found whether all targets share the same binding site on secretagogin, or whether more than one target can be bound simultaneously.

**Fig 12:**
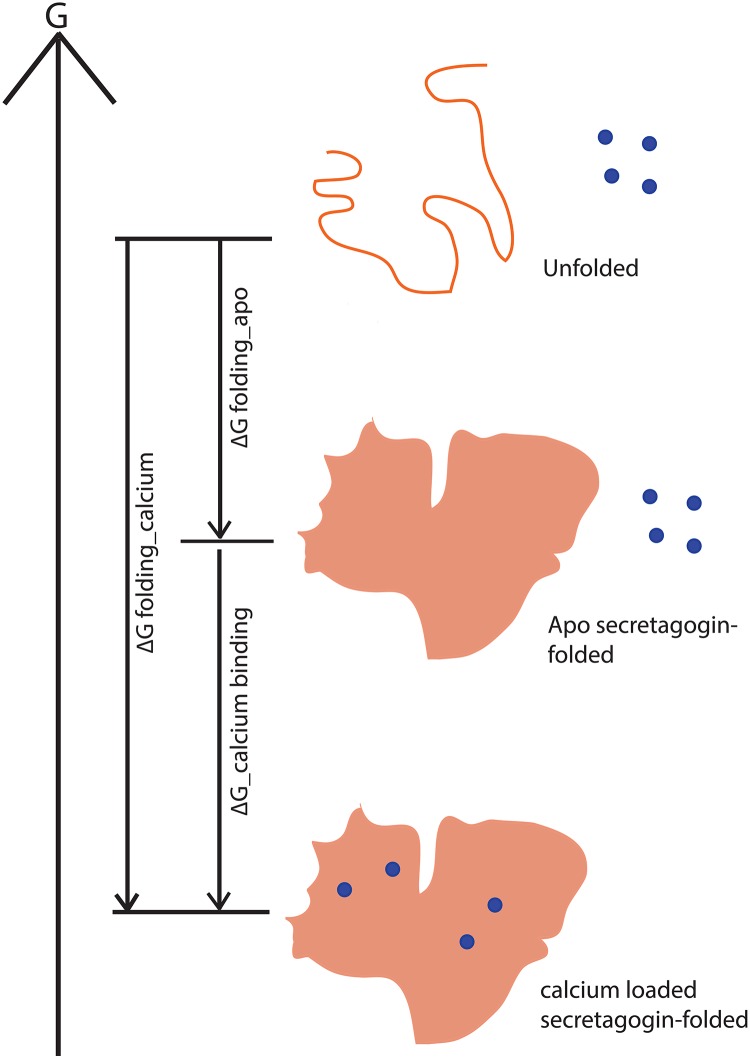
Cartoon representing the free energy changes (ΔG) involved in folding and calcium binding of an EF-hand protein. The total free energy change from its unfolded state to calcium-loaded state includes contributions from both folding and binding. Blue circles represent Ca^2+^ ions.

**Fig 13:**
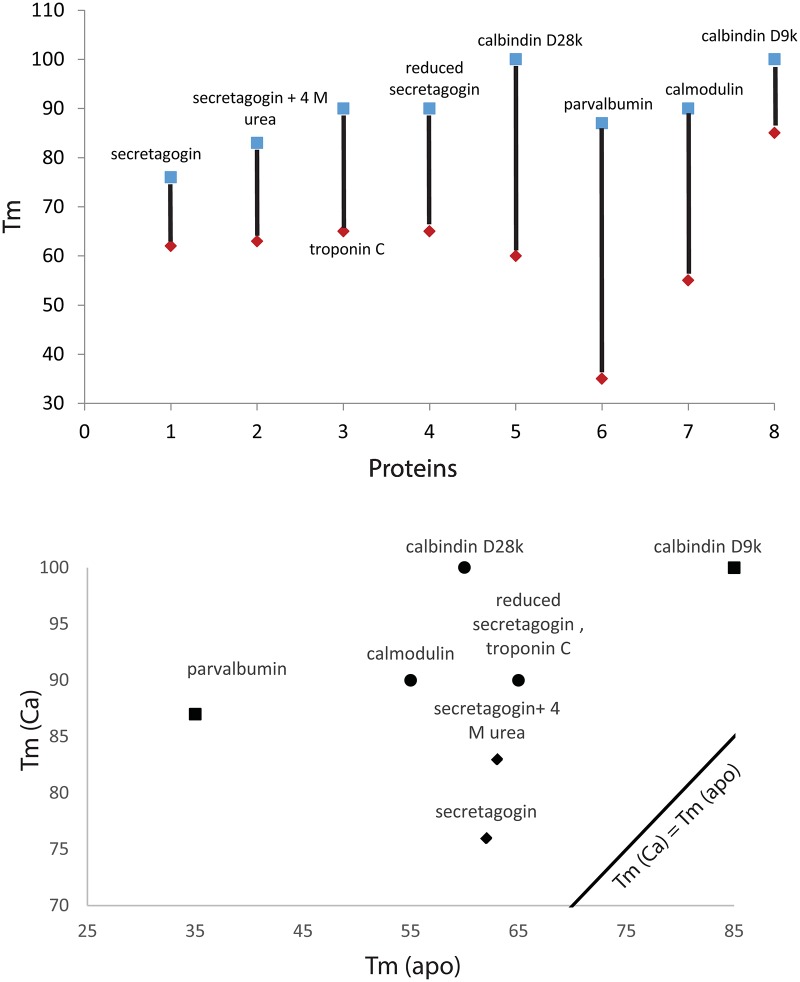
Comparison of Tm values of apo and calcium-loaded form of secretagogin and other calcium binding proteins. Apo form of protein shown in red and calcium-loaded form in blue.

The stability of secretagogin may be compared to other EF-hand calcium binding proteins like calbindin D28k, calmodulin, calbindin D9k, troponin C and parvalbumin all of which are more stable in the presence of Ca^2+^. Because of the thermodynamic linkage of binding and folding (Fig 12), the apparent stability of the Ca^2+^ form will depend on Ca^2+^ concentration. The thermal stability of secretagogin, and its Ca^2+^ dependence, compared to a panel of calcium-binding proteins is summarized in Table 2 and Fig 13. Calbindin D9k is the most stable protein on this list and apo parvalbumin the least stable. Both these proteins are found to function as Ca^2+^-buffer proteins. The proteins known to act as calcium sensors, including calmodulin, calbindin D28k and troponin C, are found in the intermediate range. All proteins in the list are considerably more stable in the presence compared to the absence of Ca^2+^. In the plot of T_m_ (Ca^2+^) vs T_m_ (apo) (Fig 13), the data for all proteins, including secretagogin, lie above the reference line Tm (Ca^2+^) = Tm (apo). Clearly, secretagogin is not as stable as calbindin D9k. Moreover, secretagogin lies closer to the reference line than the other calcium sensor proteins, meaning its stability and potentially also structure is less calcium sensitive. However, the reduced secretagogin shows higher stability, larger Ca^2+^ dependence, and the T_m_ value is closer to the Ca^2+^-buffer protein calbindin D9k. Secretagogin is thus a protein in which disulfide bonds are destabilizing rather than stabilizing the folded structure, especially in the case of the calcium-bound protein. This means that the disulfide bond is incompatible with the structure that is governed by amino acid sequence, in line with previous findings that disulfide bonds are only stabilizing if compatible with the protein structure [23, 24]. The cysteine residues in secretagogin thus have another role than protein stabilization, possibly involving redox regulation as has been found in the close homologue calbindin D28k [20].

**Table 2:** Summary of melting temperatures of secretagogin and other calcium binding proteins.

	Protein	T_m_ Ca^2+^ / °C	T_m_ apo / °C
1	(Whiting) parvalbumin[25, 26]	70	27
2	calmodulin[27]	90	55
3	calbindin D9k[28]	100	85
4	calbindin D28k[29]	100	60
5	troponin C[30]	90	65
6	secretagogin+ 4 M urea	83	63
7	secretagogin	76	62
8	reduced secretagogin	90	65

## Conclusion

Secretagogin is a relatively newly discovered hexa EF-hand protein implied in secretion and signaling processes in neurons and neuroendocrine cells. Understanding its role in these processes will require a detailed mapping of its functional as well as biophysical properties. Mapping the redox regulation and stability of the protein towards unfolding as a function of solution conditions reveals the conditions under which it is folded and monomeric or oligomeric. We find that secretagogin is a highly stable protein at physiological pH, temperature, redox potential and Ca^2+^ concentration. Moreover, under all conditions examined, calcium-bound secretagogin is more stable than apo secretagogin towards chemical and thermal denaturation. Disulfide bonds occur in secretagogin and they are relatively resistant to reduction, but this reduction destabilizes the protein towards unfolding, suggesting that the disulfide bond is not well accommodated within the most stable structure. A comparison of secretagogin with other calcium binding proteins shows that its biophysical properties are compatible with a calcium sensor function for secretagogin.

## Experimental Procedures

### Chemicals

All chemicals were of highest grade, commercially available. Six different buffer conditions were used in this study as summarized in Table 3.

**Table 3:** Various buffer conditions used to study stability of secretagogin.

Label	Buffer	CaCl_2_ or EDTA	DTT
A	10 mM Tris, 0.15 M KCl, pH 7.5	1 mM CaCl_2_	Absent
B	10 mM Tris, 0.15 M KCl, pH 7.5	0.5 mM EDTA	Absent
C	10 mM Tris, 0.15 M KCl, pH 7.5	1 mM CaCl_2_	1 mM
D	10 mM Tris, 0.15 M KCl, pH 7.5	0.5 mM EDTA	1 mM
E	10 mM Tris, 0.15 M KCl, pH 7.5	1 mM CaCl_2_	4 mM
F	10 mM Tris, 0.15 M KCl, pH 7.5	0.5 mM EDTA	4 mM

### Expression and purification of secretagogin

Human wild-type secretagogin, cloned into the PetSac vector [31], was transformed into *Escherichia coli* BL21 Star. A single colony was used to inoculate an overnight culture of 50 mL LB medium with 50 μg/mL ampicillin and 30 μg/mL chloramphenicol. The overnight culture was diluted 1:100 in 0.5 L LB medium with 50 μg/mL ampicillin and 30 μg/mL chloramphenicol. At OD600 = 0.6 protein expression was induced by addition of IPTG to a final concentration of 0.4 mM. The cells were harvested by centrifugation after 4 h and the pellet was resuspended in 10 mM Tris, 1 mM EDTA, 1 mM DTT pH 7.5 (buffer A). The sample was sonicated and centrifuged and the supernatant was applied to a 170 mL DEAE cellulose column. The column was washed with buffer A and the sample was eluted with a NaCl gradient from 0 to 450 mM in buffer A. Fractions containing secretagogin (based on agarose and SDS-PAGE electrophoresis) were pooled, diluted 1:4 in buffer A and further purified by ion exchange chromatography on an 80 mL DEAE sephacel column. A NaCl gradient from 0 to 350 mM in buffer A was used for elution. Fractions containing secretagogin were diluted 1:4 in 10 mM Tris, 1 mM EDTA, 1 mM DTT, 0.5 M NaCl pH 7.5 (buffer B) and applied to a 60 mL phenyl sepharose column for hydrophobic interaction chromatography. The column was washed with buffer B and contaminating proteins were eluted with a reverse NaCl gradient from 500 mM to 250 mM NaCl in buffer A. Secretagogin was eluted with water. The purity of secretagogin was confirmed by agarose gel electrophoresis and SDS-PAGE electrophoresis.

### Apo secretagogin (calcium free form)

Calcium free secretagogin was prepared by dialysis of sample in 10 mM Tris, 0.15 M KCl, 0.5 mM EDTA, pH: 7.5 buffer. The buffer was changed twice in 2 h before it was kept for overnight dialysis in cold room at 4°C. Fresh buffer change was done after the overnight dialysis and dialysis was continued for an hour.

### SDS-PAGE analysis of secretagogin for presence of dimers

Secretagogin samples were analyzed using SDS-PAGE electrophoresis. 1 mg/mL of secretagogin was mixed with sample buffer (0.5 M Tris HCl pH 6.8, 10% glycerol, 5% DTT (20 mM, 10 mM, 7 mM, 5 mM, 4 mM, 3 mM, 2 mM, 1mM, 0.7 mM, 0.5 mM, 0 mM), 0.05% bromophenol blue), incubated overnight at room temperature and then loaded on a 10–20% tricine gel (Invitrogen). The gel was run for 3 h at 80 V in 1x running buffer (100 mM Tris, 100 mM Tricine, 0.1% SDS, pH 8.3), stained in staining solution (0.25% Coomassie Brilliant blue, 40% ethanol, 10% acetic acid) for at least 4 h, destained in destaining solution (30% ethanol, 7% acetic acid) and finally scanned. BenchMark^™^ Low-range prestained protein standard from Novagen was used.

### SDS-PAGE and Western blotting of recombinant secretagogin oligomers in purified apo secretagogin

Purified apo secretagogin was analyzed using SDS-PAGE electrophoresis and Western blotting. Apo- secretagogin (1 mg/mL) was mixed with Laemmli sample buffer (0.5 M Tris-HCl pH 6.8, 10% (v/v) glycerol, 0.05% (w/v) bromophenol blue) and 5% (v/v) DTT at a range of concentrations (20 mM, 10 mM, 7 mM, 5 mM, 4 mM, 3 mM, 2 mM, 1 mM, 0.5 mM, 0 mM). Samples were incubated overnight at room temperature and 2 μL (2 μg) was loaded onto a 12% polyacrylamide gel. The gel was run for 1 h at 120 V in 1× running buffer (25 mM Tris-HCl pH 8.3, 192 mM glycine, 0.1% (w/v) SDS), transferred onto PVDF membrane for 90 min at 320 mA in 1× transfer buffer (48 mM Tris, 39 mM Glycine, 0.037% (w/v) SDS, 20% (v/v) methanol), and incubated overnight at 4°C with rabbit polyclonal anti-secretagogin (Sigma-Aldrich, HPA006641) diluted 1:20,000 in blocking solution (5% (w/v) skimmed milk, 10 mM Tris-HCl, 150 mM NaCl, 0.1% (v/v) Tween-20). Protein bands were visualised using chemiluminescent Western blotting substrate (Thermo Scientific) and exposing the membrane to X-ray film in the darkroom. Precision Plus Protein^™^ Dual Color Standard from BioRad was used.

### Mammalian cell maintenance and protein extraction

BRIN-BD11 rat clonal pancreatic β-cell line (kindly gifted by Bernadette Mc Evoy, UCD Conway Institute) was maintained in Roswell Park Memorial Institute medium (RPMI-1640) supplemented with 10% (v/v) foetal bovine serum, 100 UI/mL penicillin, 100 μg/mL streptomycin, and 2 mM glutamine. Cells were incubated at 37°C in a humidified atmosphere of 5% CO_2_ and 95% O_2_ and passaged in a confluence range of 1.6 × 10^6^/mL of growth medium. Cells were harvested by centrifugation at 405 *×g* for 3 min. The remaining media was aspirated and the pellet resuspended in 750 μL lysis buffer (50 mM Tris-HCl pH 7.4, 150 mM NaCl, 2 mM CaCl_2_, 1% (v/v) Triton X-100). Extracts were centrifuged at 10,000 *×g* for 10 min, 4°C to pellet cellular debris. Protein concentration was determined using binicotinicacid (BCA) assay (Thermo Scientific, Rockford, IL) as per manufacturer’s instruction. Total protein concentration was found to be 1.4 mg/mL.

### SDS-PAGE and Western blotting of secretagogin for presence of dimers in overexpressed BRIN-BD11 cells

BRIN-BD11 cells were analyzed using SDS-PAGE electrophoresis and Western blotting. Total protein (30 μg) was mixed with Laemmli sample buffer (0.5 M Tris-HCl pH 6.8, 10% (v/v) glycerol, 0.05% (w/v) bromophenol blue) and 5% (v/v) DTT at a range of concentrations (20 mM, 10 mM, 5 mM, 1 mM, 0 mM). Samples were incubated overnight at room temperature and loaded onto a 12% polyacrylamide gel. The gel was run for 1 h at 120 V in 1× running buffer (25 mM Tris-HCl pH 8.3, 192 mM glycine, 0.1% (w/v) SDS), transferred onto PVDF membrane for 90 min at 320 mA in 1× transfer buffer (48 mM Tris, 39 mM glycine, 0.037% (w/v) SDS, 20% (v/v) methanol), and incubated overnight at 4°C with rabbit polyclonal anti-secretagogin (Sigma-Aldrich, HPA006641) diluted 1:10,000 in blocking solution (5% (w/v) skimmed milk, 10 mM Tris-HCl, 150 mM NaCl, 0.1% (v/v) Tween-20). Protein bands were visualised using chemiluminescent Western blotting substrate (Thermo Scientific and Roche, as indicated) and exposing the membrane to X-ray film in the darkroom. Precision Plus Protein^™^ Dual Color Standard from BioRad was used.

### Glutathione oxidation of *E*.*coli* purified secretagogin

Secretagogin that was purified after expression in *E*.*coli* was analyzed to check if glutathione oxidation can reverse the effect of DTT reduction. A -170 mV GSSG+GSH redox buffer was made under near non-oxidizing conditions by degassing and then purging nitrogen gas into water prior to the addition of glutathione components [20]. The protein sample was reduced by 20 mM DTT and then dialyzed in the GSSG+GSH redox buffer overnight under near non-oxidizing conditions and further the sample was analyzed using SDS-PAGE electrophoresis.

### Urea denaturation of secretagogin as monitored by CD and fluorescence spectroscopy

#### a. Circular dichroism

Urea denaturation studies of secretagogin were carried out using J-815 CD spectrometer in far UV region (200–250 nm). The parameters adjusted include- two channels, one for CD and the other for HT; standard sensitivity; the digital integration time (D.I.T) of 1 s; band width of 1.00 nm; data pitch was 1.0 nm and continuous scanning mode with a scanning speed of 20 nm/ min with a single accumulation. The measurements were done at 25°C using a 1.0 mm cuvette.

0.2 mg/mL of secretagogin was prepared in six different buffers, A-F (Table 3) with concentration of urea varying from 0 M to 10 M. A 0.5 M interval was maintained from 0 M to 5 M and a 0.25 M interval from 5 M to 10 M. Urea solutions containing intermediate concentrations of urea, between 0 and 10 M, were mixed from the 0 and 10 M stock solutions. The samples were incubated overnight in cold room at 4°C and the CD spectra were measured at 25°C.

#### b. Fluorescence spectroscopy

Urea denaturation studies of secretagogin were also studied by fluorescence spectroscopy using Perkin Elmer LS 50B fluorescence spectrometer with emission spectra 300 and 450 nm and excitation fixed at 280 nm. Both excitation and emission slits were set to 5 nm; scan speed was set to 100 nm/min with two or three accumulations for each spectrum.

2 μM of secretagogin was dissolved in six different buffers, A-F (Table 3) with various concentrations of urea 0 M to 10 M with 0.5 M interval from 0 M to 5 M and 0.25 M interval from 5 M to 10 M and intermediate concentrations of urea, between 0 and 10 M, were mixed from the 0 and 10 M stock solutions. The samples were incubated overnight in cold room at 4°C before the measurement of spectra at 25°C.

The urea denaturation curves obtained were analyzed using Kaleidagraph software. The equation used to fit observed ellipticity, the wavelength of maximum fluorescence emission (λmax), Y_o_ was

$$Y_{0}=\frac{\left((Y_{N})+(Y_{U})e^{\frac{{{}^{-(\Delta }}^{G}{}^{{}_{NU}-m_{D}[D]}}{RT}}\right)}{\left(1+e^{\frac{{{}^{-(\Delta }}^{G}{}^{{}_{NU}-m_{D}[D]}}{RT}}\right)}$$ (1)

where Y_N_ and Y_U_ are the baselines before and after the actual unfolding. These were assumed to have zero slope, because rather short baselines were observed. ΔG_NU_ is the unfolding free energy in pure buffer, m_D_ is the influence of denaturation concentration on the stability, R is the molar gas constant and T is the temperature. The free energy toward unfolding by urea, ΔG_NU_, is assumed to obey linear relation:

$$\Delta G_{NU}=\Delta G_{NU}(H_{2}\text{O})-m_{D}[D]$$ (2)

C_m_, the urea concentration at the transition midpoint, was calculated from Eq 2 by setting ΔG_NU_ to zero.

### Temperature denaturation as monitored by CD spectroscopy

The secretagogin samples with no urea, 1 M urea or 4 M urea were prepared in six different buffers A-F (Table 3) and subjected to temperature denaturation. A start temperature of 20°C and end temperature of 95°C was used at a scan rate 1°C/min and a reverse scan from 95°C to 20°C was performed afterwards. The general settings include two channels, one for CD and the other for HT; standard sensitivity, digital integration time of 16 s; band width of 2.00 nm and wavelength was set to 222 nm. Samples with 0.2 mg/mL secretagogin in 0 M, 1 M or 4 M urea samples were prepared exactly in the same way as for urea denaturation. The samples were incubated overnight at 4°C before starting the CD measurement.

#### Temperature denaturation reversibility tests

As the temperature denaturation of secretagogin is irreversible when the protein is heated to 95°C, tests were performed to find the maximum temperature from which the protein can return to its native fold. As earlier, apo or calcium-loaded secretagogin in no urea, 1 M urea or 4 M urea were prepared in six different buffers A-F (Table 3). For apo secretagogin 45, 55 and 65°C were tested and for calcium-loaded secretagogin 55, 65 and 75°C were tested in all buffers. For each test temperature, a scan was first recorded from 20°C to the test temperature and then a reverse scan back to 20°C. Depending on the results, additional intermediate temperatures were tested. This was done to check out the closest temperature range for renaturation to occur.

## Supporting Information

S1 Fig Freshly prepared and gel filtered secretagogin sample from *E*.*coli* in 10 mM Tris, 0.15 M KCl, 0.5 mM EDTA, pH: 7.5. (A) UV-absorbance peak of gel filtration of secretagogin sample. Arrows 1–8 on the peak indicates sample collected at different points. (B) 1–8 samples collected in A is loaded on SDS-PAGE gel.
(TIF)

S2 Fig apo (A) vs calcium-loaded (B) secretagogin treated with 0–20 mM DTT and analyzed immediately on SDS-PAGE electrophoresis. (TIF)

## Abbreviations

CaBP: calcium binding protein
CD: circular dichroism
Cm: concentration of urea at transition mid-point
DEAE: diethyl amino ethyl cellulose
DTT: dithiothrietol
IPTG: isopropyl β-D-1-thiogalactopyranoside
m_D_: influence of denaturation concentration on the stability
OD: optical density
PAGE: poly acrylamide gel electrophoresis
SDS: sodium do-decyl sulfate
Tm: melting temperature
Tm apo: melting temperature of apo form of protein
Tm Ca: melting temperature of calcium-loaded protein
ΔG_NU_: unfolding free energy in pure buffer
λ max: wavelength with maximum fluorescence intensity
[Θ]_222 nm_: ellipticity at 222 nm
