# Supplementary figures and images for: Calcium Binding and Disulfide Bonds Regulate the Stability of Secretagogin towards Thermal and Urea Denaturation
Source: PLoS One. 2016 Nov 3;11(11):e0165709. doi: 10.1371/journal.pone.0165709 (PMC5094748; doi:10.1371/journal.pone.0165709)

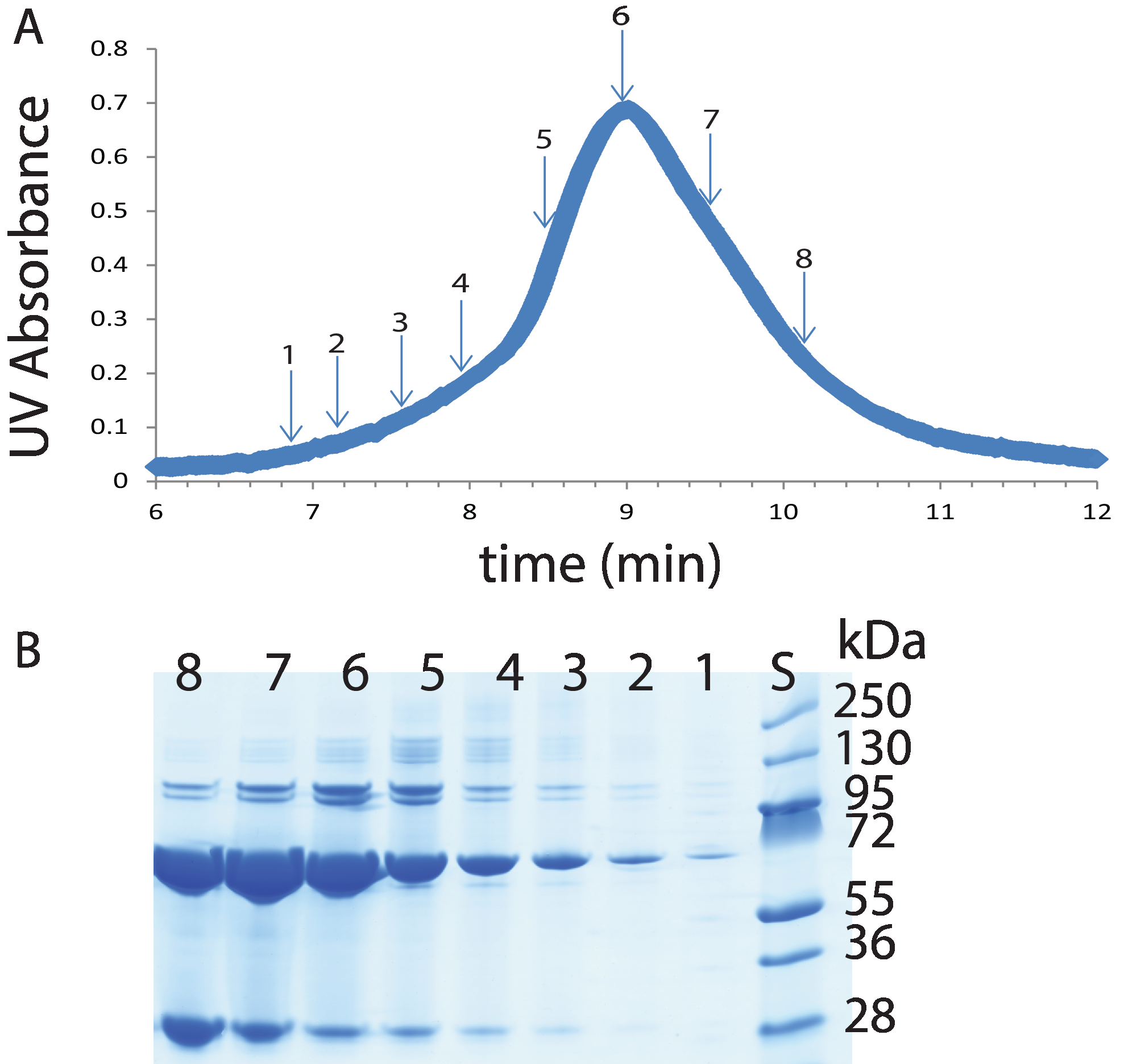

Supplement: S1 Fig — (A) UV-absorbance peak of gel filtration of secretagogin sample. Arrows 1–8 on the peak indicates sample collected at different points. (B) 1–8 samples collected in A is loaded on SDS-PAGE gel. (TIF) [file pone.0165709.s001.tif]

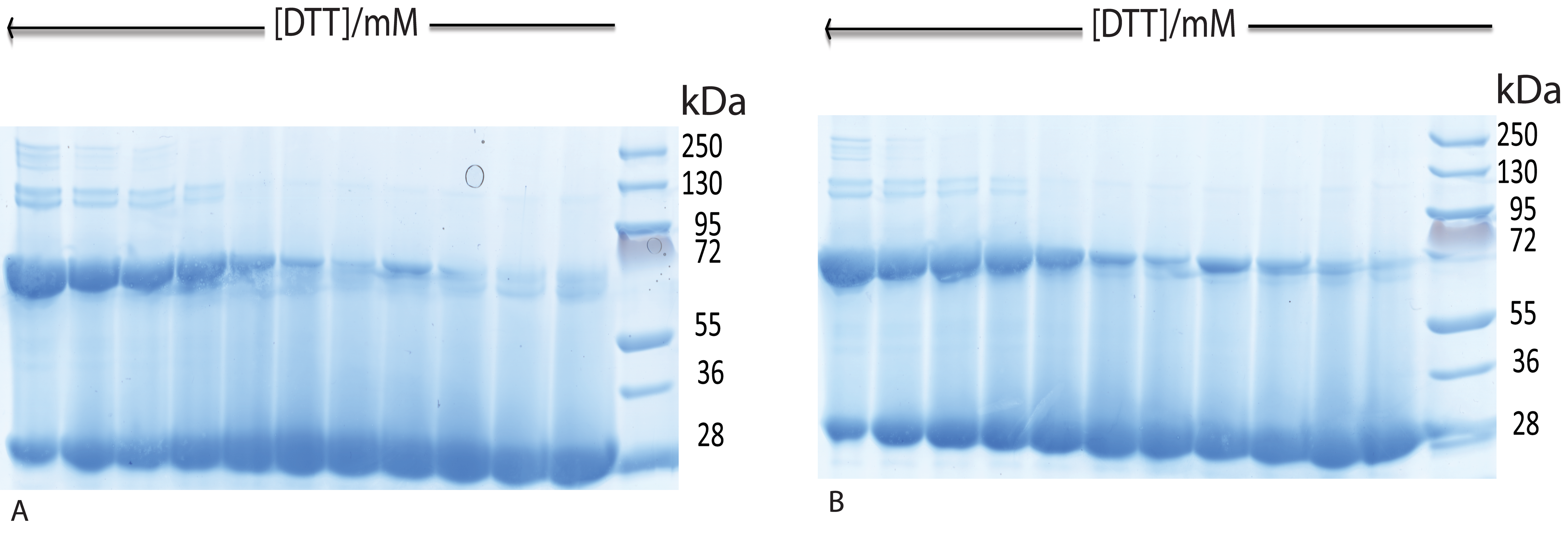

Supplement: S2 Fig — (TIF) [file pone.0165709.s002.tif]
